# Seismic surveys reduce cetacean sightings across a large marine ecosystem

**DOI:** 10.1038/s41598-019-55500-4

**Published:** 2019-12-16

**Authors:** A. S. Kavanagh, M. Nykänen, W. Hunt, N. Richardson, M. J. Jessopp

**Affiliations:** 1 MaREI Centre, Environmental Research Institute, University College Cork, Beaufort Building, Ringaskiddy Co. Cork, P43C573 Ireland; 2 School of Biological, Earth and Environmental Sciences, Environmental Research Institute, University College Cork, Co. Cork, T23N73K Ireland; 3 Marine Institute, Rinville, Galway, T23N73K Ireland

**Keywords:** Behavioural ecology, Conservation biology, Ecosystem ecology

## Abstract

Noise pollution is increasing globally, and as oceans are excellent conductors of sound, this is a major concern for marine species reliant on sound for key life functions. Loud, impulsive sounds from seismic surveys have been associated with impacts on many marine taxa including mammals, crustaceans, cephalopods, and fish. However, impacts across large spatial scales or multiple species are rarely considered. We modelled over 8,000 hours of cetacean survey data across a large marine ecosystem covering > 880,000 km^2^ to investigate the effect of seismic surveys on baleen and toothed whales. We found a significant effect of seismic activity across multiple species and habitats, with an 88% (82–92%) decrease in sightings of baleen whales, and a 53% (41–63%) decrease in sightings of toothed whales during active seismic surveys when compared to control surveys. Significantly fewer sightings of toothed whales also occurred during active versus inactive airgun periods of seismic surveys, although some species-specific response to noise was observed. This study provides strong evidence of multi-species impacts from seismic survey noise on cetaceans. Given the global proliferation of seismic surveys and large propagation distances of airgun noise, our results highlight the large-scale impacts that marine species are currently facing.

## Introduction

Anthropogenic activities influence marine ecosystems in every part of the world’s oceans^1^, and marine species have long been exposed to their detrimental effects^2^. Over 60% of the global oceans experienced increases in human impacts in the five years leading up to 2015^1^. Within this context, impacts from underwater noise pollution have been recognized as significant environmental issue^3^, with human activities such as commercial shipping, seismic surveys, and sonar being major sources of marine noise^4^. Airguns fired aboard seismic survey vessels are used for seabed mapping and hydrocarbon exploration, and produce pulsed, high intensity sounds. Sound source levels of 248–255 dB re 1 µPa-m, zero-to-peak, are typical of full scale seismic arrays, with levels of at least 10 dB less when measured horizontally around vessels compared to downward directed sounds^5^. Global spending on seismic data acquisition has been steadily increasing over the past 20 years, with almost US$10 billion spent on marine seismic surveys in 2013 alone^6^. Despite this method of exploration being commonly employed globally for over 40 years, more knowledge is needed on its broad-scale ecological impacts.

The impacts of seismic survey noise, at scales immediately surrounding the sound source, have been recorded in a diverse range of species, including fish, invertebrates, plankton, and reptiles^7–11^. However, the low frequency sounds generated by seismic airguns, commonly below 200 Hz^12^, may extend over large distances, particularly in deeper waters^13^. These sounds have been recorded at locations up to 4,000 km from the source^14^, and can ‘blanket’ areas of up to 300,000 km^2^ with noise^15^. Generally, the dominant frequencies of seismic airguns overlap with those of the communication signals of baleen whales (10 Hz–1 kHz) and seismic sounds may be audible to toothed whales at distances up to 100 km away^5^. However, due to the varying propagation characteristics of sound in water, the frequencies of sounds and received noise levels may differ in surface waters and with distance from the source^16,17^, and recorded impacts vary greatly^18^.

All cetacean species rely on acoustic signaling for important life functions, such as communication, orientation, or locating prey, raising concerns about the potential impacts of seismic airgun noise on these animals in particular^18,19^. Available evidence suggests that seismic survey noise may influence the behaviour of cetaceans in a number of ways^18,20^ potentially leading to reduced sighting rates e.g.^21^. Long- and short-term displacement, changes in vocal, diving and movement behaviour of baleen whales species have all been recorded in response to seismic surveys^22–28^. Similar effects have also been noted by studies focused on toothed whale species; including displacement of sperm whales (*Physeter macrocephalus*), short-term displacement of harbour porpoise (*Phocoena phocoena*) close to the noise source, and avoidance behaviours by Atlantic spotted dolphin (*Stenella frontalis*) in response to seismic noise^29–31^. Notwithstanding this growing body of evidence supporting the negative effects of seismic surveys on cetaceans, most studies to date have focused on individual species, were carried out at relatively small spatial or temporal scales, and often lacked non-seismic-control data to accurately determine impacts^21,23,28,30–32^.

The Northeast Atlantic is a diverse marine environment both in term of habitat and species richness, and is home to over 40 species of marine mammal^33^, many of which are considered either endangered, threatened or data deficient^34^. These animals are likely attracted to areas of significant biological productivity such as the Celtic-Biscay Shelf Large Marine Ecosystem (LME)^35^. Over the last ten years, substantial levels of seismic survey activity have been carried out across much of this area, providing an opportunity to examine the effect of seismic activity on acoustically sensitive marine mammals over a broad and diverse spatial scale. The aim of this study was, therefore, to investigate the effect of seismic surveys, and in particular, airgun activity, on the sighting density of cetaceans (both baleen and toothed whales) across multiple species and habitats. To do this we used cetacean sightings data collected on seismic vessels during operations, and comparable data collected from control (non-seismic) vessels. Cetacean sightings were modelled using an additive Generalised Estimating Equations-Complex Region Spatial Smoother (GEE-CReSS) model that included temporal and environmental variables in addition to a spatial component. This study is the first to combine cetacean surveys from seismic survey vessels with other cetacean surveys as control data, taking a multi-species, large-scale approach to examining the effect of seismic surveys on cetaceans, while also accounting for the potential influence of spatial, environmental and temporal variables.

## Results

**Figure 1:**
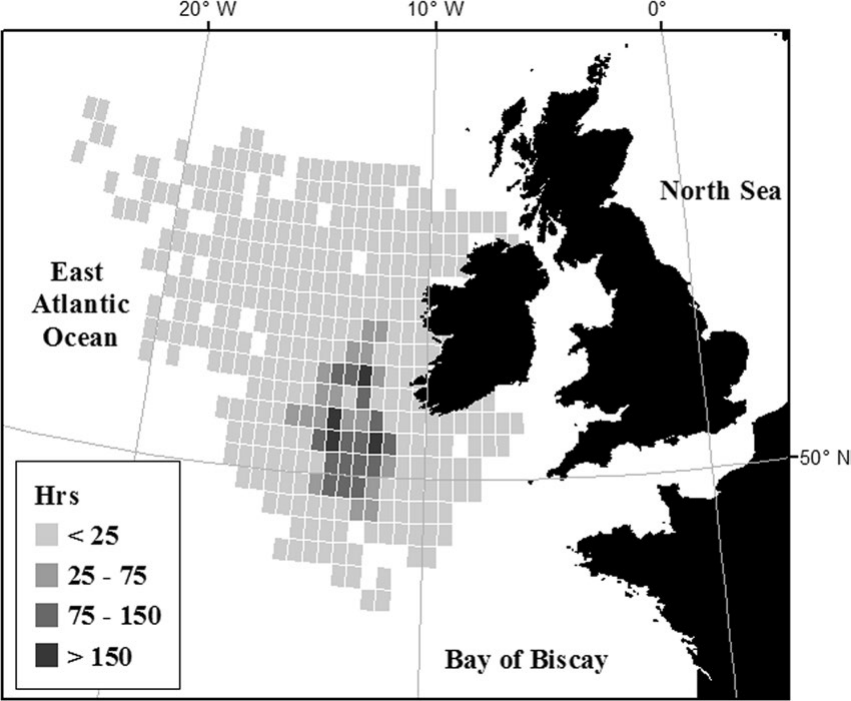
Distribution of all survey effort on a 0.5° × 0.5° grid.

Approximately 8,000 hrs of survey effort data was incorporated in the analysis (Fig. 1) including 1,020 hrs of control data and 6,980 hrs of seismic data (seismic data did not include pre-shooting, soft-starts, ramp-up or airgun testing periods) (Fig. 1, Supplementary Fig. S1). Baleen whale species were recorded on 395 occasions, and toothed whale species on 538 occasions during seismic surveys (active and inactive periods combined), and on 96 and 311 occasions during control surveys.

**Figure 2:**
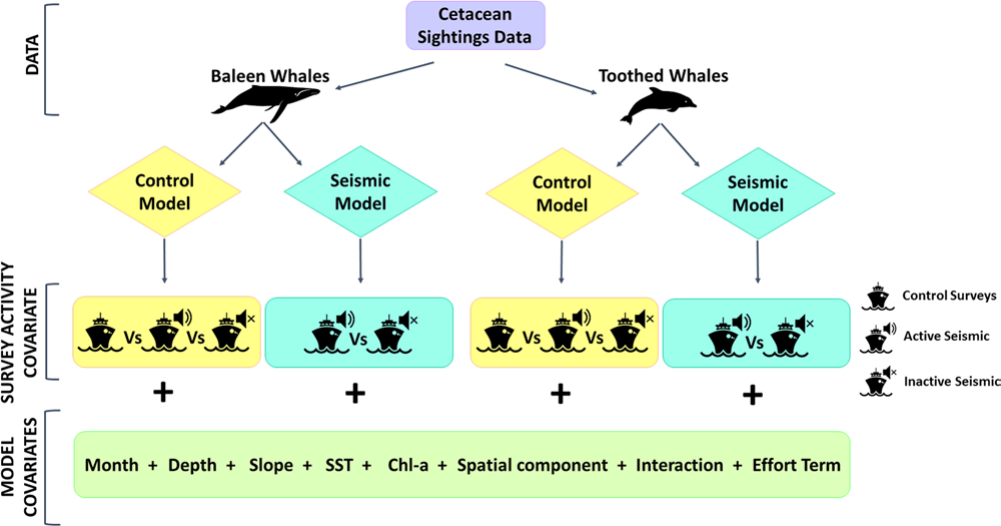
Summary of the modelling approach used to estimate the effect of seismic survey noise on the baleen whale and toothed whale sighting density. For the control models, ‘survey activity’ included three levels; active, inactive, and control (no airgun activity on a non-seismic vessel); the level ‘control’ was incorporated in the models as baseline. For the seismic models, the factor covariate ‘survey activity’ included two levels: active (airguns on operating on full-power on a seismic survey vessel), inactive (no airgun activity on a seismic survey vessel); the level ‘active’ was used as the baseline. The covariate ‘interaction’ is the interaction of the spatial component and survey activity, and the ‘effort term’ is the effective search area that was included in the models to account for different amount of survey effort. Whale, dolphin, ship, and speaker icons were sourced from the Noun project (https://thenounproject.com) and used unchanged under Creative Commons CCBY (https://creativecommons.org/licenses/by/3.0/us/legalcode). Whale icon by Philipp Lehmann, dolphin icon by Aleks, Ship icon by Juliette Design, and speaker icon by Diego Naïve.

Four different models were employed to assess the effect of airgun activity and survey type on the cetacean sighting density (Fig. 2). Control models compared active and inactive periods of seismic surveys with control surveys, while seismic models compared active periods with inactive periods within seismic surveys. Control and seismic models were carried out for baleen and toothed whales separately. All models accounted for temporal, spatial and environmental factors that may have simultaneously influenced cetacean detectability and a summary of the different sighting density models run can be found in Fig. 2 (see also Table 1, Supplementary Figs. S2 and S3).

**Table 1:** Summary of control and seismic model results.

			Survey Activity		Month	Depth	Slope	SST	Chl-a	Spatial	Interaction
			Inactive	Active							
(1)	Control	Baleen Whale	↓ (87)	↓ (88)	+	+	+	+	+	+	
(2)	Control	Toothed Whale	↓ (29)	↓ (53)	+		+		+	+	
(3)	Seismic	Baleen Whale	ns	NA	+		+			+	+*
(4)	Seismic	Toothed Whale	**↑** (35)	NA	+	+			+	+	

For the ‘survey activity’ covariate, a ↓ symbol indicates a significant negative model coefficient (*P* < 0.05), i.e. a significant decrease in cetacean sightings, a ↑ symbol indicates a significant positive model coefficient (*P* < 0.05) i.e. a significant increase in cetacean sightings, and ‘ns’ indicates a non-significant result. Reduction in sighting density (compared to the baseline) are presented in brackets. All other covariates retained in the best GEE-CReSS control and seismic models are indicated with a plus sign (+), and their effect on cetacean sightings densities are presented as partial residual plots in Supplementary Figs. S2 and S3. Control baleen whale model (1), control toothed whale model (2), seismic baleen whale model (3), seismic toothed whale model (4). For the control models, ‘survey activity’ included three levels; active, inactive, and control (no airgun activity on a non-seismic vessel), the level ‘control’ was used as the baseline. For the seismic models, the factor covariate ‘survey activity’ included two levels: active (airguns on operating on full-power on a seismic survey vessel), inactive (no airgun activity on a seismic survey vessel), the level ‘active’ was used as the baseline. The covariate ‘spatial’ denotes the spatial component consisting of x and y coordinates, the covariate ‘interaction’ indicates an interaction of the spatial component and survey activity (an asterisk * indicates a significant interaction variable).

### Control models (comparison of active and inactive seismic surveys with control surveys)

**Figure 3:**
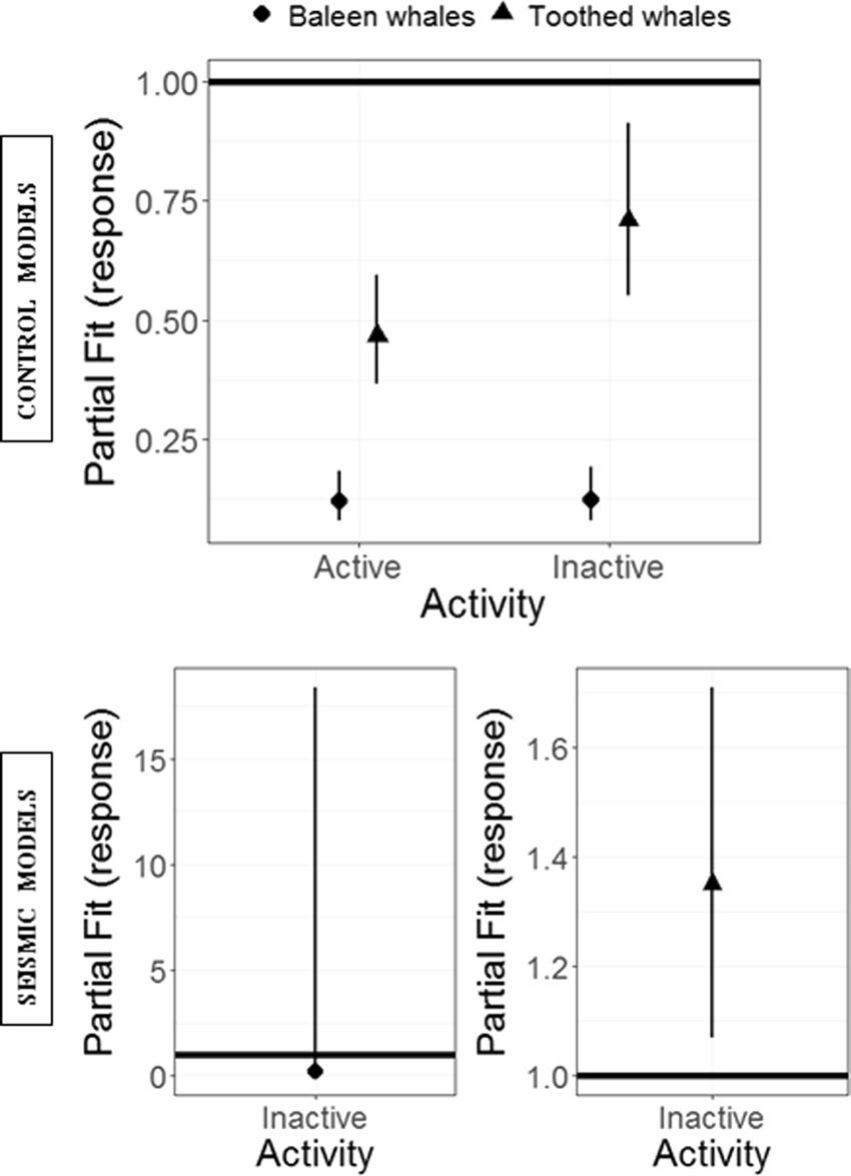
The effect of seismic surveys on cetacean sighting densities represented as partial residual plots with 95% confidence interval for the factor ‘activity’ in the control models, and in the seismic models, for baleen whales (filled circles) and toothed whales (filled triangles). In the control models, the factor level control was used as the baseline (horizontal line in the plots); in the seismic models, the factor level active was used as the baseline.

**Figure 4:**
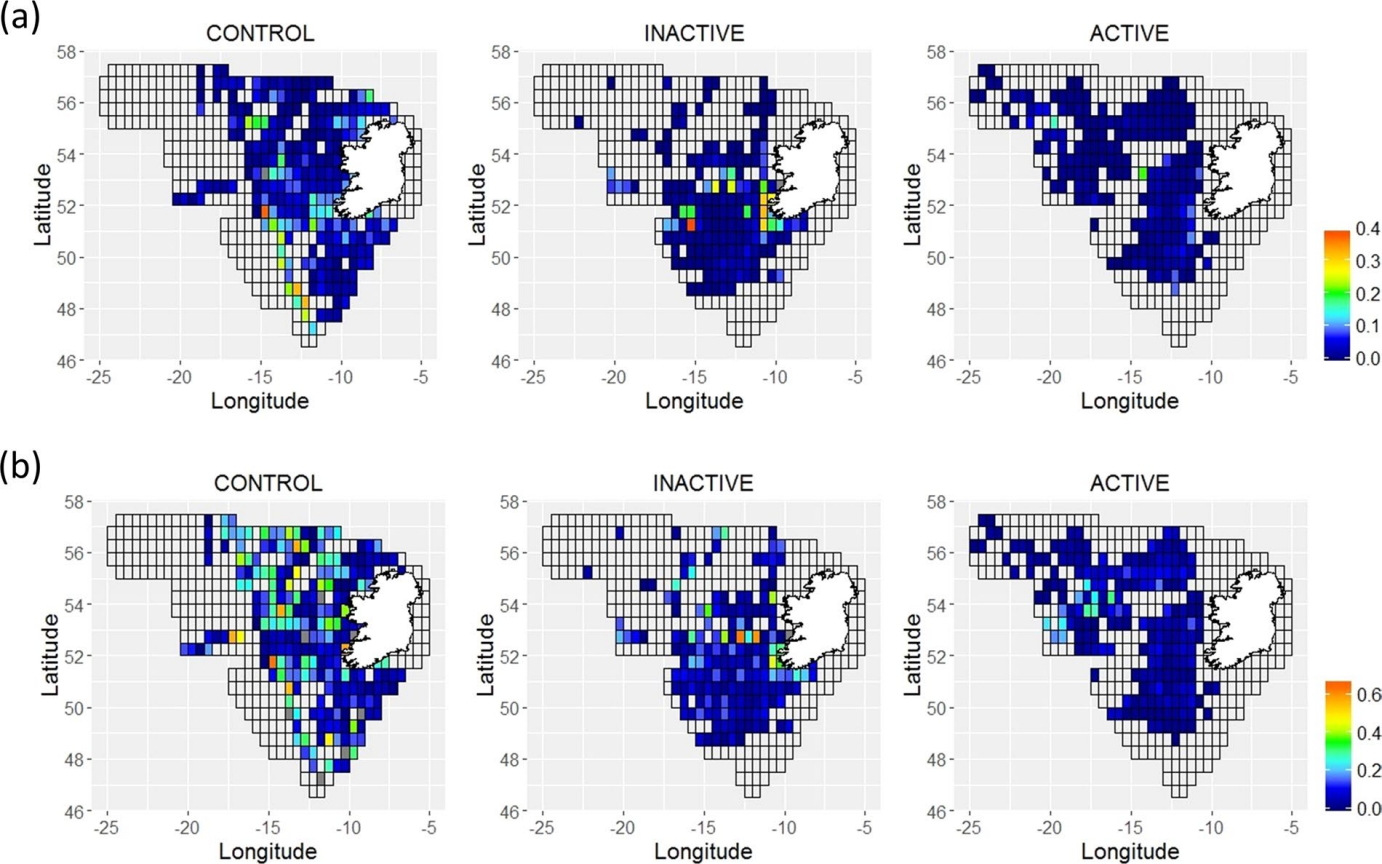
Model predicted mean sighting densities per grid cell with control models for (**a**) baleen and (**b**) toothed whales. The mean sighting density varies from low (blue) to high (red). 95% confidence intervals for predicted densities are presented in the supplementary material (Supplementary Figs. S6 and S7).

Control models highlight a significant effect of seismic surveys on sighting densities of both baleen and toothed whales (Tables 1 and 2). When compared to control surveys, and when keeping all the other model covariates constant, baleen whale sighting densities across the study site were reduced by an average of 88% (95% CI: 82–92%) and 87% (95% CI: 81–92%) during active and inactive airgun periods, respectively (both *P* < 0.0001, Tables 1 and 2, Fig. 3). Toothed whale sighting densities were reduced by an average of 53% (95% CI: 41–63%) and 29% (95% CI: 9–45%) during active and inactive airgun periods (*P* < 0.0001, *P* < 0.001) when compared to control surveys (Tables 1 and 2, Fig. 3). The best fitting baleen and toothed whale models included the spatial covariate as a significant predictor; however, the interaction of the spatial covariate and seismic activity was not retained in either model. These results imply an overall effect on sightings density, with fewer animals detected during seismic surveys, independent of geographic location (Fig. 4). Uncertainty around the sighting density model predictions are provided in supplementary materials (Figs. S6 and S7).

**Table 2:** Coefficients of the ‘survey activity’ for each GEE-CReSS model on the response scale, their standard error (SE), and P-values.

	Model		Covariate	Coefficient	SE	Wald’s P
(1)	Control	Baleen Whale	Active	0.1200	0.0259	<0.0001
			Inactive	0.1230	0.0282	<0.0001
(2)	Control	Toothed Whale	Active	0.4670	0.0570	<0.0001
			Inactive	0.7090	0.0913	0.0300
(3)	Seismic	Baleen Whale	Inactive	0.1890	0.4420	0.5078
(4)	Seismic	Toothed Whale	Inactive	1.3500	0.1610	0.0298

Control baleen whale model (1), control toothed whale model (2), seismic baleen whale model (3), seismic toothed whale model (4). Covariate levels are described in Table 1.

### Seismic models (comparison of active periods with inactive periods of seismic surveys)

Seismic models showed no difference in baleen whale sighting density between active and inactive airgun periods (*P* = 0.5078, Fig. 3); however, the best model included the interaction of the spatial component and seismic activity, which was significant (*P* < 0.0001), (Tables 1 and 2) indicating a reduction in sighting densities in some areas whilst other areas remained unaffected or experienced an increase (Fig. S8). Seismic models highlighted a significant difference in sighting densities of toothed whales between active and inactive airgun periods, with 35% more sightings during inactive periods (*P* = 0.0298) (Tables 1 and 2, Fig. 3). Similar to the control model results, the best seismic model included the spatial covariate as a significant predictor of the number of sightings and did not include the interaction with seismic activity. Again, this indicates an overall reduction in sighting densities of animals during seismic surveys that is independent of location (Fig. S9).

### The effect of highly represented species

Fin whales (*Balaenoptera physalus*) were the most commonly sighted baleen whale species, while common dolphins (*Delphinus delphis*) were the most regularly encountered toothed whale species (Supplementary Table S2). The removal of these highly represented species had no effect on the results of the baleen whale control and seismic models; however, there was some evidence for species-specific responses to seismic survey activity for common dolphins (Table 3). The significant reduction in toothed whale sighting densities between active and inactive airgun periods was retained in the seismic models without common dolphins, whilst no significant difference was recorded for common dolphins only (seismic models). No significant difference between inactive and control periods was noted in control models without common dolphins, or for common dolphins only (Table 3).

**Table 3:** GEE-CReSS model coefficients on the response scale, their standard error (SE), and *P*-values, of the ‘survey activity’ covariate for control and seismic models after removing highly represented species.

Model		Covariate	Coefficient	SE	Wald’s P
(a)	Baleen Whale (no FW)	Active	0.1627	0.0366	<0.0001
		Inactive	0.1574	0.0404	<0.0001
(b)	Toothed Whale (no CD)	Active	0.4957	0.0798	0.0002
		Inactive	0.9244	0.1613	0.6963
(c)	CD only	Active	0.5557	0.1218	0.0182
		Inactive	0.7108	0.1497	0.1492
(d)	Baleen Whale (no FW)	Inactive	0.2784	0.2751	0.1416
(e)	Toothed Whale (no CD)	Inactive	1.7800	0.2720	0.0008
(f)	CD only	Inactive	1.0600	0.1910	0.7280

Control baleen whale model (without fin whale data) (a), control toothed whale model (without common dolphin data) (b), control model common dolphin data only (c), seismic baleen whale model (without fin whale data) (d), seismic toothed whale model (without common dolphin data) (e), seismic model common dolphin data only (f). Insufficient data were available to run control and seismic models for fin whale data only. Covariate levels are described in Table 1. Species codes: fin whale (FW), common dolphin (CD).

## Discussion

The world’s oceans cover over 70% of the earth’s surface, contain more than 90% of the earth’s water, and are estimated to be home to 2.2 million species^36^. As a result, the effects of increasing anthropogenic disturbance in this vast environment is rapidly becoming an ecological issue of global significance. Against a backdrop of increasing anthropogenic disturbance in our oceans, dwindling fossil fuel reserves are driving the search for hydrocarbon resources further offshore. Therefore, assessing the impact of seismic surveys across diverse and relatively inaccessible habitats is of great importance. This study examined the effect of seismic surveys on cetaceans in an extensive and bathymetrically diverse offshore region of the Northeast Atlantic. Our results suggest a significant effect of seismic surveys across multiple cetacean species, with consistent decrease in sightings, particularly in the offshore waters to the west and southwest of Ireland, an area identified as of relative importance for cetaceans^37^.

Baleen whales are generally assumed to be more vulnerable to disturbance from seismic airgun noise than toothed whales due to a greater overlap with the frequency ranges of their communication signals and hearing sensitivities^5^. However, we also noted a similar reduction in toothed whale sightings across species and habitats in this study. Although very little information on the seasonality of cetacean distribution is available for offshore Eastern Atlantic waters, current literature suggests a low temporal overlap between seismic surveys (concentrated between June and August) and the timing of peak occurrences of baleen whales in our study LME^38^. Despite this, the results of the baleen whale control model found a significant reduction in sightings associated with seismic activity, and the seismic model indicated that although the overall sighting densities of baleen whales remained unaffected, some redistribution of these animals occurred. In contrast to baleen whales, the peak occurrences of many offshore toothed whales may coincide more closely with the peak in seismic activity in this study^38^. As a result of this temporal overlap, toothed whales may be more regularly exposed to seismic noise than baleen whales in the Northeast Atlantic, and for longer time periods, potentially resulting in the significant reduction in sightings densities observed in this study. With the exception of common dolphins, highly represented species did not influence overall model outputs suggesting that while there may be variability in the thresholds required to elicit a behavioural response and in the spatial/temporal scale of impact, the response of cetaceans to seismic survey noise is somewhat ubiquitous. While our results are consistent with previous findings of localised spatial avoidance^21,22^, and temporary displacement^29,30^ of whale species in response to seismic noise, they also highlight the importance of timing of the seismic activity in mitigating against the disturbance.

While we noted a significant difference between control surveys and both active and inactive airgun periods of seismic surveys, we found no effect of airguns on baleen whale sightings within seismic surveys (active *versus* inactive periods). While this may be indicative of a temporal effect relating to species-specific movement or susceptibility to disturbance as inactive airgun periods often occur between active periods, we argue that they cannot be considered representative of baseline conditions and should not be used as a control when assessing impacts of seismic surveys. The scale and scope of this study allowed us to examine the effects of seismic surveys on cetaceans across a large marine ecosystem whilst accounting for spatial and environmental factors, and conclusively found reduced cetacean sightings as a result of seismic survey activity. However, the limitations of the available data are such that although differences in detectability resulting from distance between the survey vessel and the animals were accounted for, low overall sighting numbers meant that it was not possible to calculate detectability at the level of individual observers, or to account availability bias of the animals (i.e. the proportion of time spent submerged and therefore unavailable for detection). Furthermore, the data are unsuited for quantifying the spatio-temporal scale of impacts outside the visual range and observation periods of the observers, or to conclusively identify the nature of the effect, i.e. displacement from the area or a behavioural change (for example decreased surfacing rates) resulting in reduced detectability. While detection functions for seismic surveys showed higher probability of detection further from the trackline than for control surveys, whether this is a behavioural response due to cetaceans avoiding areas of highest noise along the trackline remains speculative.

Sound propagation models for seismic survey noise accounting for bathymetry, sediment, water temperature/salinity and ambient noise, show that the noise ‘footprint’ (i.e. the area where seismic survey noise frequencies detectable by cetaceans above ambient noise) of seismic surveys is potentially large in deep waters^13^, and can be at levels consistent with temporary threshold shifts and behavioural responses in marine mammals^39,40^. In addition, studies have shown that airgun activity along the continental margins propagates into the deep ocean and is a significant component of low-frequency noise^14^. While permanent hearing threshold shifts are less likely to occur at ranges beyond 500 m from the sound source^39,41^, it is likely that received noise levels may exceed thresholds for eliciting behavioural responses in cetaceans over much larger distances than the visual range of observers on survey vessels. However, more research is needed to specifically quantify species-specific behavioural response thresholds under different social and environmental conditions and determine the spatio-temporal scale of the effect. The increasing global coverage^6^, large noise propagation distances^13,14^, and documented impacts of seismic airgun noise across a range of vertebrate and invertebrate species^7–11^ suggests that seismic survey effects on marine biota are likely to increase. This must be taken into account when assessing species distributions and designating protected areas for them, especially when species are facing additional threats relating to climate change and the loss of habitat.

## Methods

Marine Mammal Observer (MMO) data from seismic and control vessels was collected in an area encompassing approximately 880,000 km^2^ of the Atlantic, between 16°W and 5°W, and 57°N and 48°N (Fig. 1). The area contains a diverse variety of habitats, including shallow shelf waters approximately 200 m in depth, underwater sea cliffs and canyon systems, deep water environments such as troughs, and abyssal plains which reach depths of over 4000 m. Over the last ten years there have been substantial sea floor mapping surveys, as well as exploration for oil and gas deposits in the Northeast Atlantic (Supplementary Fig. S1). Seismic survey vessels carry MMOs as part of mitigation practices. MMOs undertake visual surveys for cetaceans prior to the commencement of operations (the start of air-gun generated noise), and regularly continue surveying throughout seismic operations. However, inactive periods during seismic surveys are generally surrounded by active periods, and as a result cannot be considered a true baseline of cetacean encounter rates. Therefore, between April 2015 and July 2017 a control data acquisition programme was undertaken (Supplementary Table S1). MMOs were deployed opportunistically on research vessels to undertake surveys for cetaceans in areas covered also by seismic surveys (Supplementary Fig. S1).

Survey methodologies employed by both survey types (seismic and control) followed Joint Nature Conservation Committee (JNCC) survey guidelines and datasheets^42^. A single experienced observer, located on the viewing platform of the survey vessel, scanned out to the horizon with the naked eye for presence of marine mammals. Binoculars with reticles or range finding sticks were used to estimate distances from the survey vessel and confirm sightings or species identifications. MMOs were at similar heights onboard both seismic and control vessels (mean seismic vessel observer height 14.37 m ± 2.96 SD, mean control vessel observer height 14.74 m ± 2.64 SD). Seismic observers scanned a 360° zone around the vessel, while control observers scanned a 180° zone forward of the vessel, with the difference accounted for within the effort term (offset in the sighting density models) described below. Observers recorded the GPS track of the vessel, the duration of all survey effort periods, and sightings data. Survey data was quality checked and any observations missing accurate vessel locations, observer effort, cetacean id or group size were not included in the analysis. This was more likely to occur in older surveys where less strict reporting requirements were in place. A summary of seismic and control surveys is provided in the supplementary material (Supplementary Table S1).

During seismic survey pre-shooting periods lasting for 30–60 minutes depending on water depth^42^, observers focused on an ‘exclusion zone’ of 500 m. Due to the methodological differences between this pre-shooting monitoring and operational seismic and control monitoring pre-shooting periods were omitted from analysis. Similarly, seismic soft-start and ramp up periods, where airguns are not operating at full capacity, were not included in the seismic data set.

### Environmental variables

Four environmental variables were included in the sighting density models; water depth (m), seabed slope angle (°), sea surface temperature (SST) measured in °C, and chlorophyll-*a* (Chl-a) measured in mg/m^3^. Environmental variables, such as those chosen here, are often considered useful proxies for ocean productivity or prey availability. Depth and slope data were derived from the INFOMAR bathymetry map (https://www.gsi.ie/en-ie/data-and-maps/Pages/Marine.aspx); average values for each line of survey effort in each cell in the 0.5° × 0.5° grid of Irish waters were extracted using the Spatial Analyst tool of ArcGIS 10.2 (ESRI, 2014). SST and Chl-a were extracted as monthly composites, at a resolution of 4 km, from the SeaWiFS website^43^. Average monthly SST and Chl-a values were calculated for each cell across the grid of Irish waters in ArcGIS.

### Sightings

For each cetacean sighting the following information was recorded: (i) species or species group (where species-level identification was not possible, species list given in Supplementary Table S2), (ii) position of the vessel, (iii) the distance and bearing to sighting, (iv) the behaviour of animals, (v) weather conditions, and (vi) the ‘activity’ of the vessel (active or inactive airguns, soft starts/ramp-ups, and periods of airgun testing in the case of seismic surveys). For seismic surveys analysis, only data where airguns were operating at full power (active), or were completely inactive (excluding pre-shooting periods), were included in the analyses. Baleen and toothed whale sightings were analysed separately based on the different hearing sensitivities^40^ and differences in the visual detectability of these groups (Fig. S4).

### Imperfect detection and survey effort

To account for imperfect detectability of cetaceans due to, for example, distance to the animal(s), group size and weather conditions, detection functions were fitted to sightings data using the angle and distance to sightings in order to calculate distance to the survey trackline in the R package *Distance*^44,45^ (Supplementary Fig. S5). Detection functions were generated separately for each survey type (seismic and control) and cetacean group (baleen and toothed whales), with sightings truncated to maximum distance of 7000 m. This truncation distance was chosen to maximize the data available for inclusion in the subsequent models for cetacean sighting density. Each of the four detection function global models included the covariates for Beaufort sea state, group size, and individual survey ID (an ID assigned to each individual control and seismic survey) to account for inherent differences in detectability between survey types (seismic/control), individual surveys (i.e. individual vessel/observer combinations), and in different weather conditions. Model fit was confirmed using standard goodness-of-fit tests for distance sampling, and hazard or half-normal detection function and variable selection was based on Akaike’s Information Criterion (AIC) scores (all three covariates were retained in all four final models). These detection functions were then used to weight the effort term in the sighting density models (see below).

Sea state specific Effective Strip Widths (ESWs)^46^ were derived from detection function models for baleen and toothed whales, for each individual survey, with a single exception; insufficient baleen whale sightings were recorded to calculate survey-specific ESWs for control surveys, in this case sea state specific ESWs were generated for baleen whales for all control surveys combined. An Effective Search Area (ESA), used in the sighting density models (see below) as an offset to account for different amount of effort between the different survey types, for each survey leg was calculated as 2 × r × 1 where r is the appropriate ESW (individual survey, survey type and sea state specific for that effort leg) and l is distance travelled during the survey leg. The resulting effort term is a measure of area surveyed in km^2^.

### Modelling cetacean sightings densities

Cetacean sightings (baleen and toothed whales examined separately) were modelled using an additive GEE-CReSS model^47,48^ with a quasi-Poisson error distribution for over-dispersed count data (number of sightings). Previous studies have used inactive airgun periods as a control for the effect of active airguns. However, inactive periods are often surrounded by active airguns, such that inactive periods may have already been subject to behavioural responses such as movement away from the area. We specifically tested for this by using control vessels as a baseline for cetacean occurrence. However, we were also interested in differences in sightings rates between active and inactive airgun periods. We therefore performed two analyses; one comparing data from seismic survey vessels and non-seismic vessels (control models), and another comparing active and inactive periods of seismic surveys (seismic models). The models included one factor covariate, survey activity, which included two levels for seismic models; active (airguns operating on full-power on a seismic survey vessel), and inactive (no airgun activity on a seismic survey vessel), and three levels for the control models; active, inactive, and control (no airgun activity on a non-seismic vessel) (see Fig. 2). The models also included a temporal covariate (month), and environmental covariates, depth (m), seabed slope angle (°), SST (°C), chlorophyll-*a* (mg/m^3^), and a spatial element consisting of an interaction between x and y coordinates of the study grid projected in Universal Transverse Mercator (UTM). An effort term of the effective search area (km^2^) (ESA) was included in each model as an offset. The ESA was calculated from sea state and vessel specific detection functions for baleen and toothed whales, for each survey type, and is described in detail in section Imperfect detection and survey effort.

Multi-collinearity of model covariates was tested by calculating Generalised Variation Inflation Factors, GVIFs, and GVIF^1/(2⋅df)^ ^49–51^, where df is degrees of freedom. Selecting terms based on GVIF^1/(2⋅df)^ enables the comparison of GVIFs across dimensions (i.e. factors and continuous covariates)^51^, and covariates with GVIF^1/(2⋅df)^ > 1.5 were dropped from the models. Autocorrelation function plots were used to visually check the level of any spatial or temporal autocorrelation in the model residuals, and generalised estimating equations^52^ were subsequently applied with the purpose of explicitly modelling the observed autocorrelation^53^. With this approach, data points were divided into independent blocks constituting of each grid cell, day and survey activity, and a correlation structure for the residuals was specified within the blocks^52^.

The R package ‘MRSea’^48^ with Spatially Adaptive Local Smoothing Algorithm (SALSA)^54^ and CReSS^47^ was used to fit splines to the continuous covariates in the model. SALSA is an automated procedure that finds the best way to fit a regression spline for one or two-dimensional covariates and performs knot selection, or otherwise reduces the covariate to a linear term^48,54^. Quadratic B-splines were used to model the continuous covariates, month, depth, slope, SST and chlorophyll-*a*. The first step involved running the model including only the one-dimensional covariates without the spatial element with the R-function ‘runSALSA1D_withremoval’. This function uses Quasi-likelihood under the model independence criterion (QIC) values in the selection of one-dimensional terms dropping terms automatically, after which the best model is run with the two-dimensional spatial component using CReSS. The final selection for the best model involves comparing the cross-validation (CV) scores from the model including only the one-dimensional covariates and both one- and two-dimensional terms, and the GEE-element is added to the chosen model with the lowest CV score. Repeated Wald’s tests were used to assess the significance of the retained covariates, which are presented in Table 1. The model coefficients were transformed onto response scale and the associated standard errors were calculated using the delta-method. Finally, diagnostic residual plots were inspected to assess the fit of the best models, and predictions were made on the number of sightings during different survey types and phases of seismic activity using the MRSea function ‘preds-cress’ and plotted. The confidence intervals for the predictions were obtained with 1000 bootstraps.

### The effect of highly represented species

Cetacean species were not evenly represented within the baleen and toothed whale datasets (Supplementary Table S2). Therefore, to investigate if highly represented species had a disproportionate effect on the results, the seismic and control models were re-run excluding fin whales and common dolphins as well as including only these species.

## Supplementary information

Supplementary Information

Supplementary Information

## Data Availability

All seismic survey data is publicly available through the Department of Communications, Climate Action and Environment (DCCAE), Ireland (http://www.dccae.gov.ie/en-ie/Pages/home.aspx#). The control dataset generated during the current study is available through the National Biodiversity Data Centre (NBDC), Ireland (http://www.biodiversityireland.ie/). The complete dataset and code will also be made available on Dryad prior to publication.
