# Supplementary material for: Seismic surveys reduce cetacean sightings across a large marine ecosystem
Source: Sci Rep. 2019 Dec 16;9:19164. doi: 10.1038/s41598-019-55500-4 (PMC6915703; doi:10.1038/s41598-019-55500-4)
Supplement: Supplementary file 1 — Supplementary Information [file 41598_2019_55500_MOESM1_ESM.pdf]

Seismic surveys reduce cetacean sightings across a large marine ecosystem

Supplementary Information

A.S. Kavanagh, M. Nykänen, W. Hunt, N. Richardson, M. Jessopp

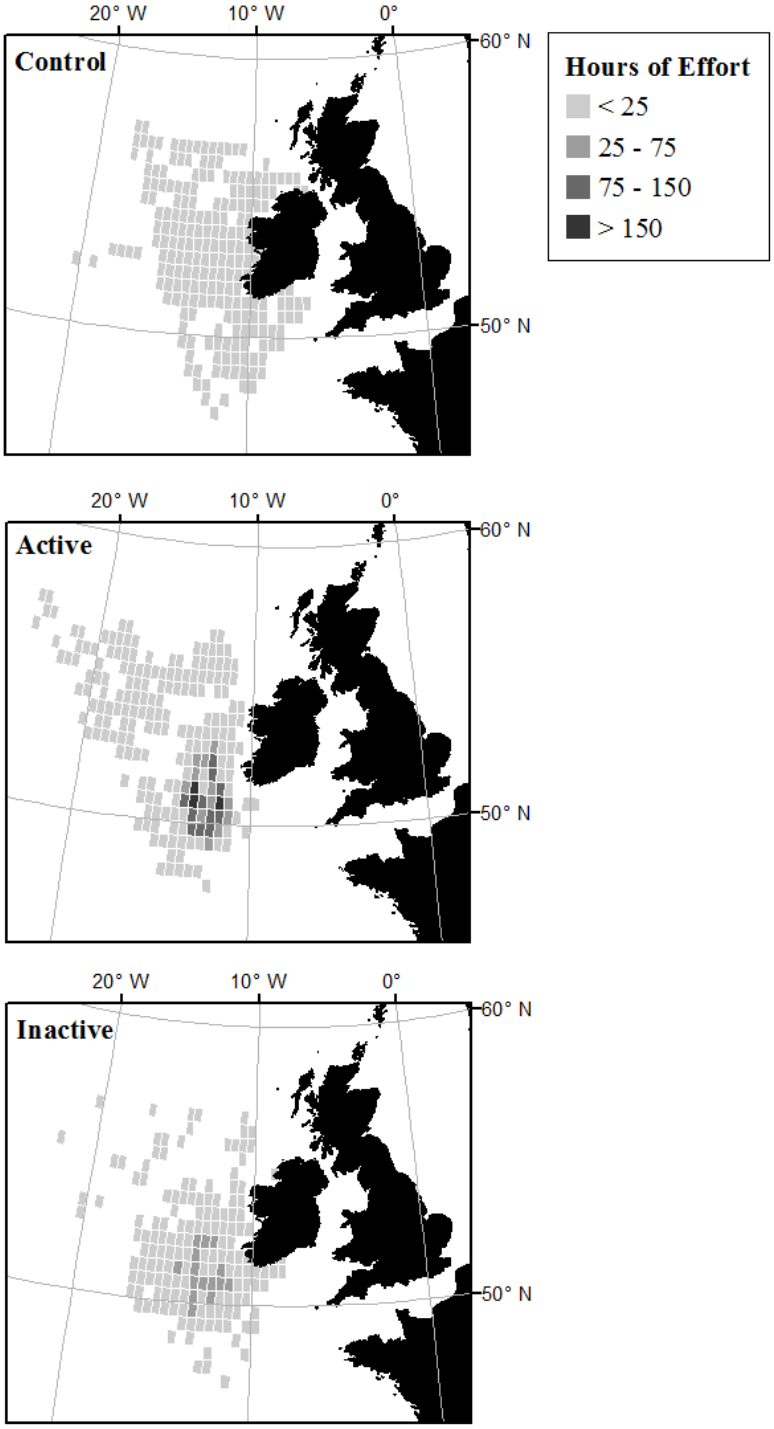

Fig. S1: Maps of survey effort on a 0.5° x 0.5° grid for control surveys and seismic surveys (Active and Inactive periods), in sea state ≤ 4 for the study area.

(a)

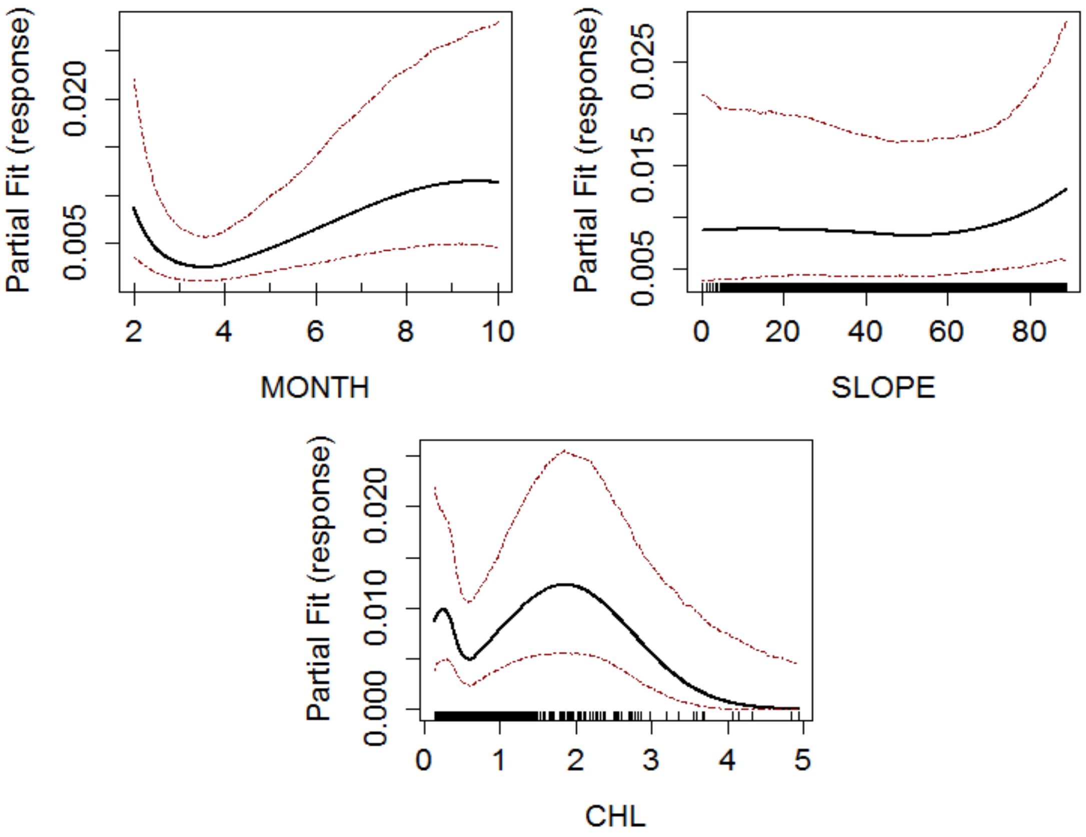
Partial Fit (response)
0.005
0.020
2
4
6
8
10
MONTH
Partial Fit (response)
0.005
0.015
0.025
0
20
40
60
80
SLOPE
Partial Fit (response)
0.000
0.010
0.020
0
1
2
3
4
5
CHL

(b)

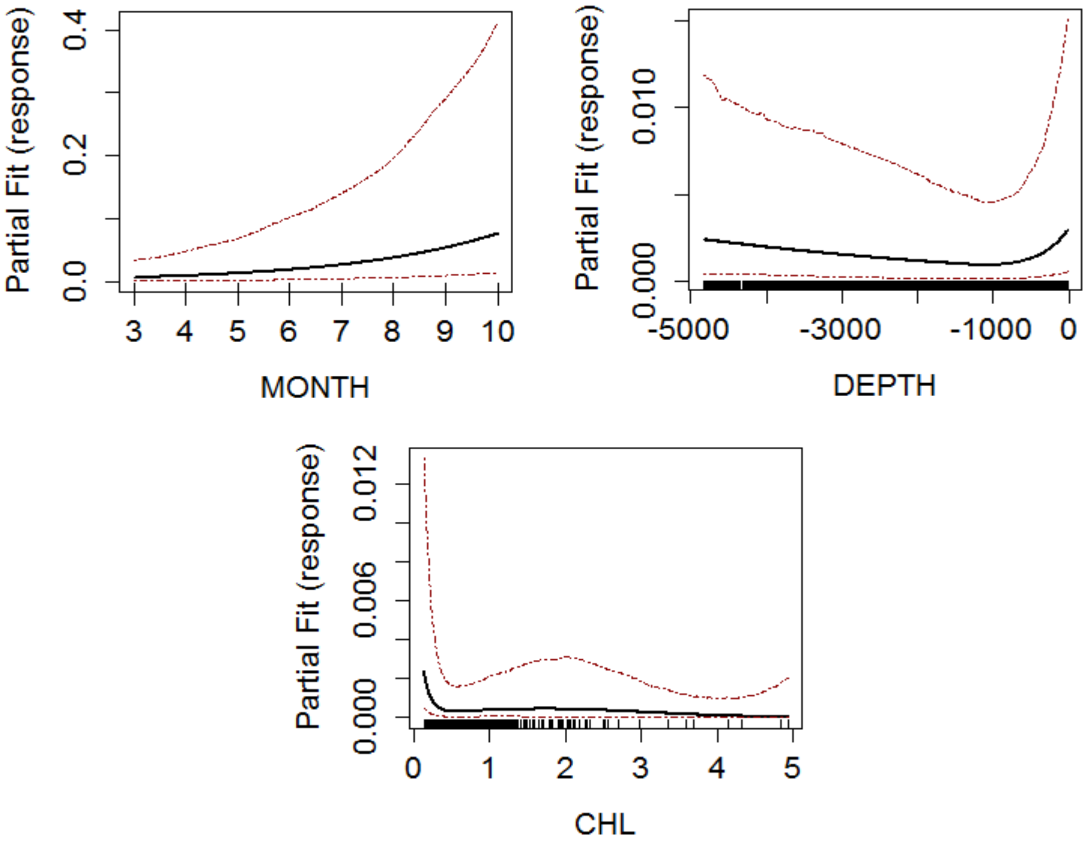
Partial Fit (response)
0.0
0.2
0.4
3
4
5
6
7
8
9
10
MONTH
Partial Fit (response)
0.000
0.010
-5000
-3000
-1000
0
DEPTH
Partial Fit (response)
0.000
0.006
0.012
0
1
2
3
4
5
CHL

Fig. S2: The effect of the continuous covariates retained in the (a) control models and (b) seismic models for toothed whale sighting densities represented as partial residual plots. The solid line is the mean and the dotted lines the 95% confidence interval. The rug plot on the x-axis shows the actual data values.

(a)

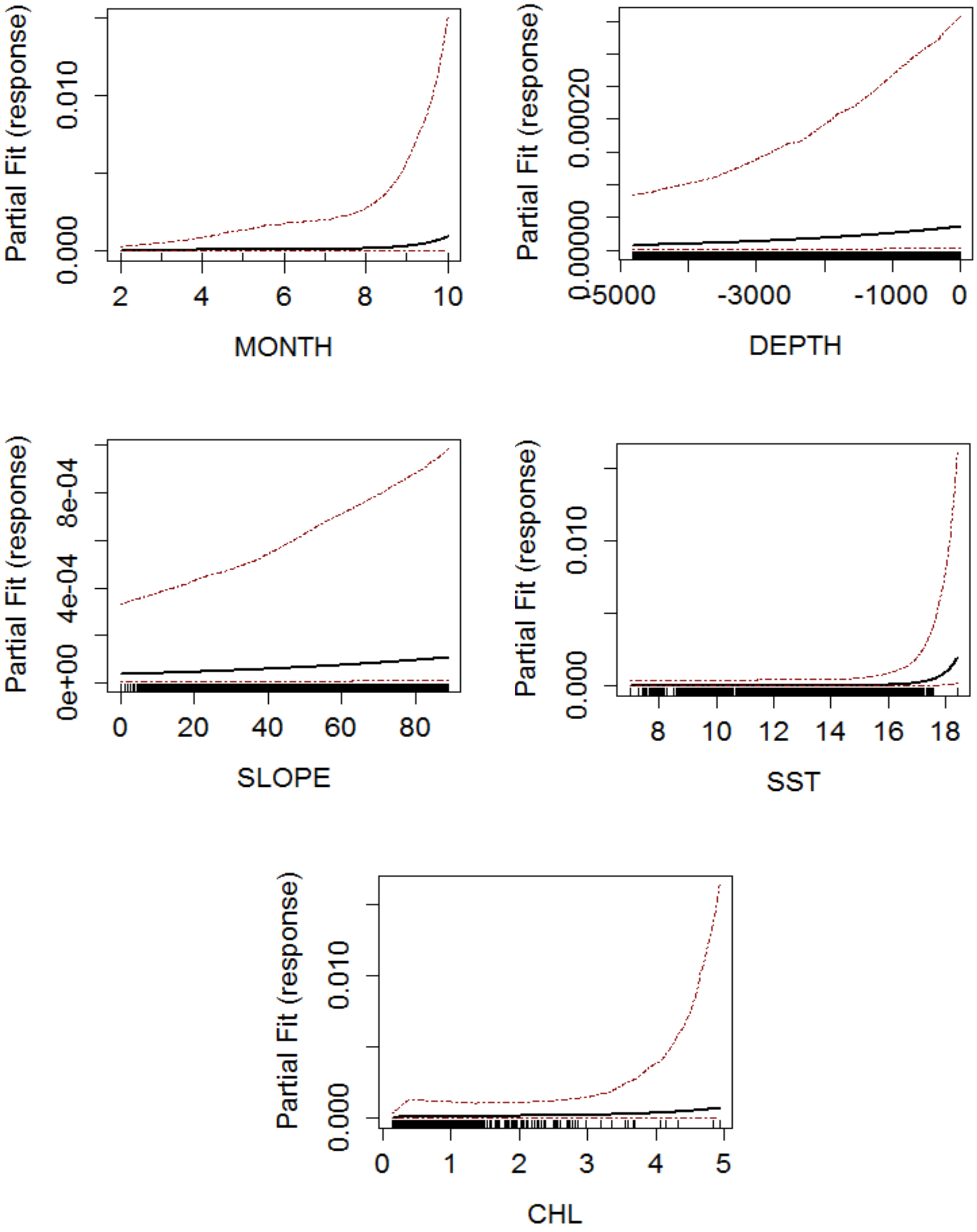

Partial Fit (response)
0.000
0.010
2
4
6
8
10
MONTH
Partial Fit (response)
0.00000
0.00020
-5000
-3000
-1000
0
DEPTH
Partial Fit (response)
0e+00
4e-04
8e-04
0
20
40
60
80
SLOPE
Partial Fit (response)
0.000
0.010
8
10
12
14
16
18
SST
Partial Fit (response)
0.000
0.010
0
1
2
3
4
5
CHL

(b)

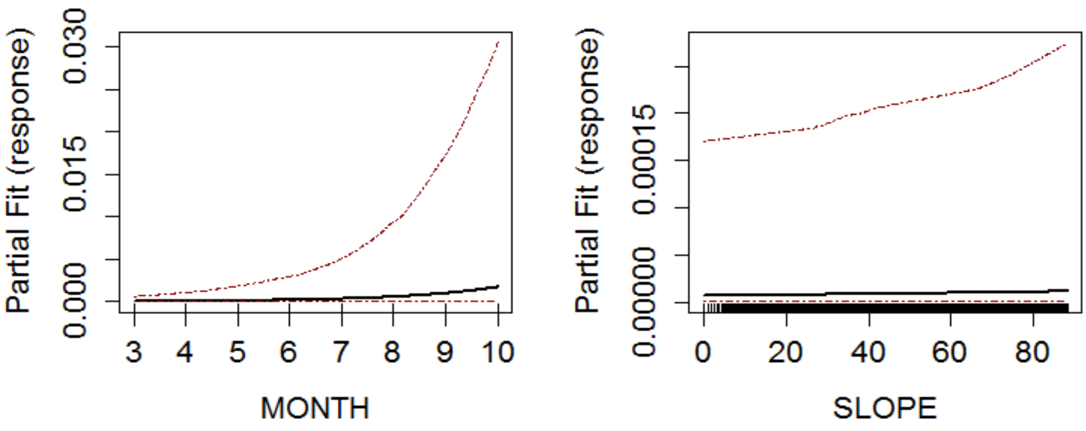

Fig. S3: The effect of the continuous covariates retained in the (a) control models and (b) seismic models for baleen whale sighting densities represented as partial residual plots. The solid line is the mean and the dotted lines the 95% confidence interval. The rug plot on the x-axis shows the actual data values.

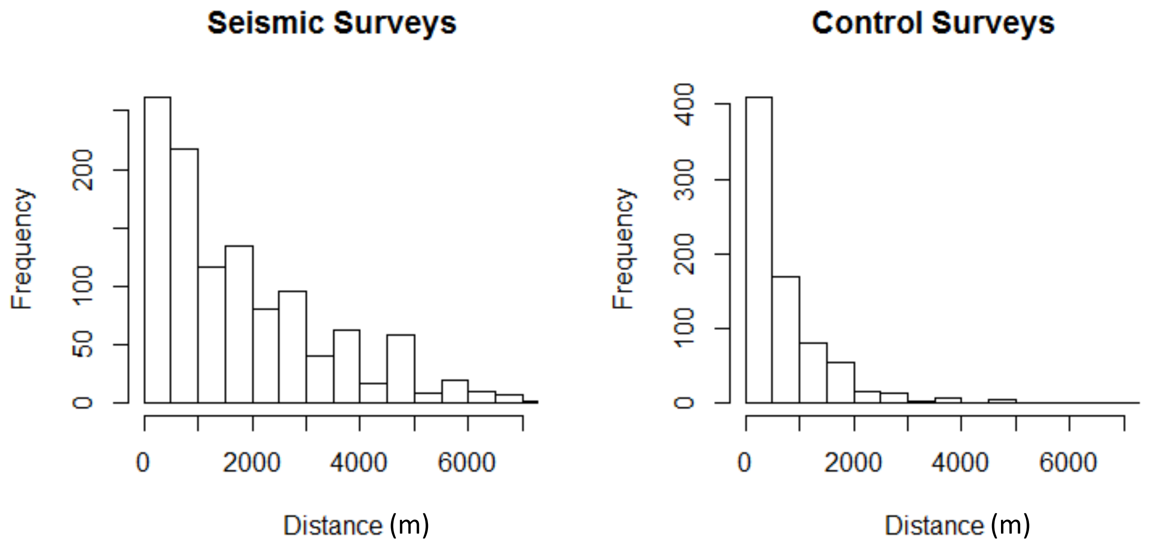

Fig. S4: Histograms of sighting distances for seismic surveys (active and inactive periods combined) and control surveys truncated to 7000 m.

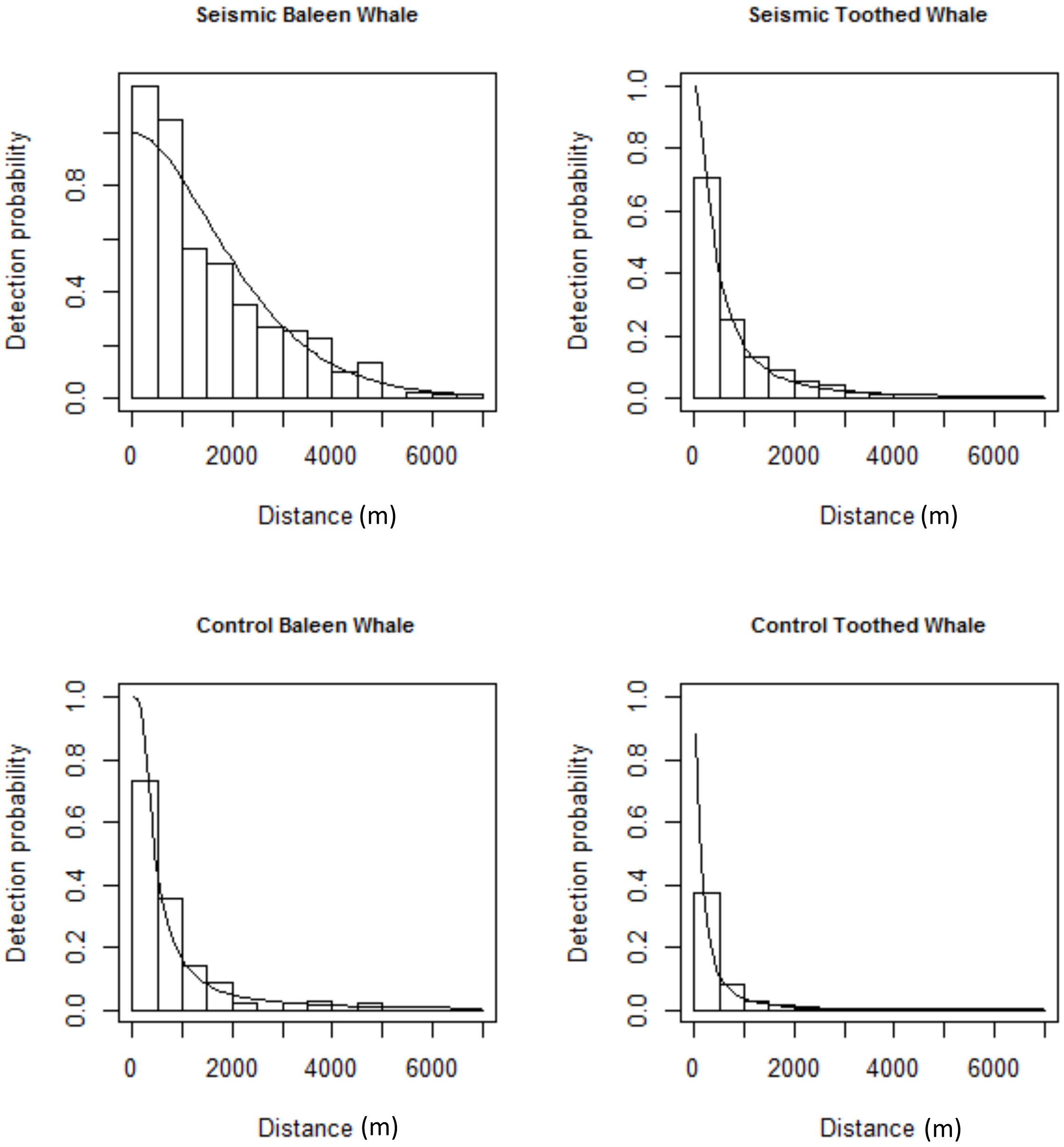

Fig. S5: Fitted detection functions to the seismic and control data from baleen and toothed whales.

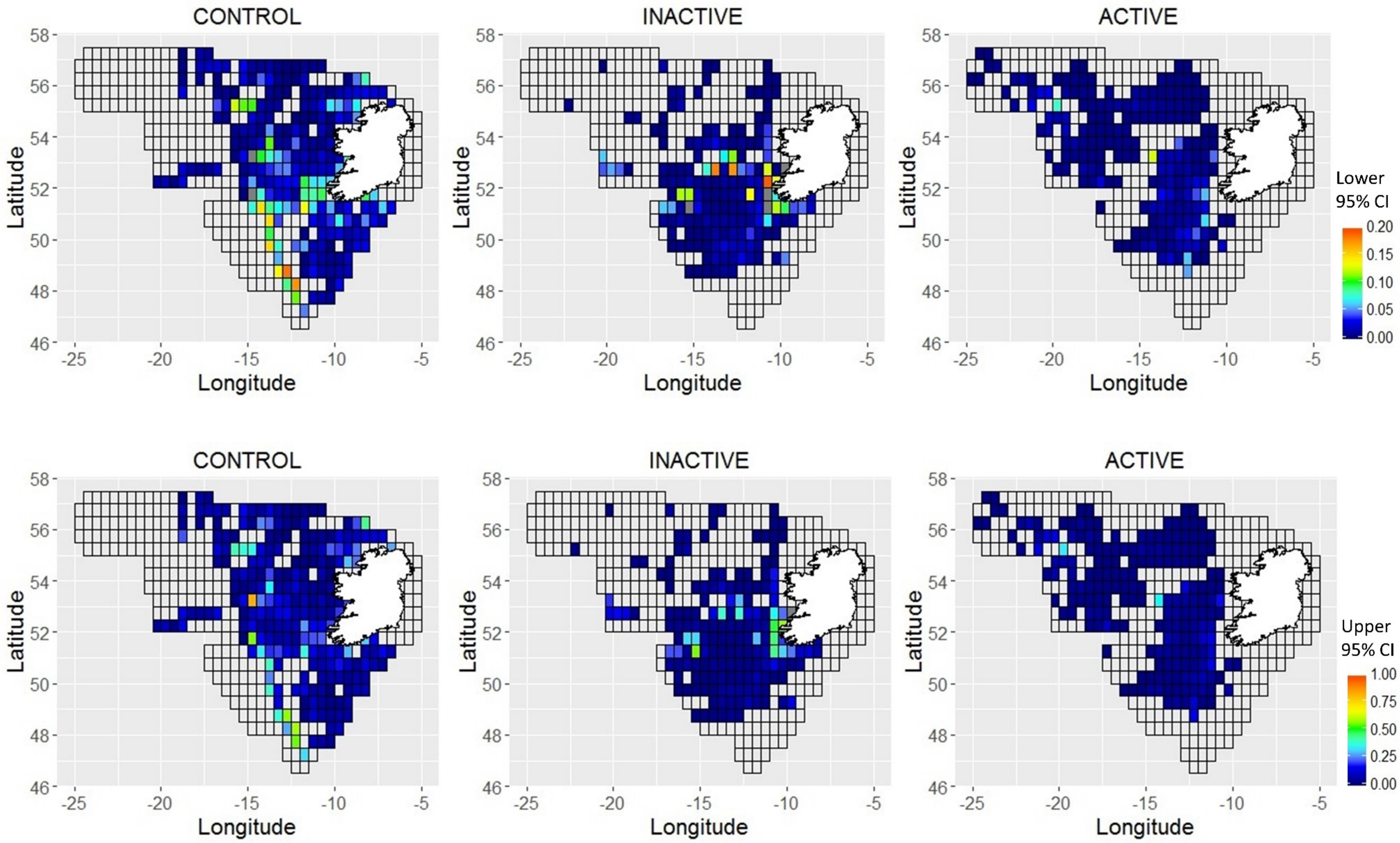

Fig. S6: Model predicted 95% confidence interval (CI) of sighting densities (per grid cell) for baleen whales in the control model (i.e. the effect of inactive and active seismic surveys compared to the control surveys).

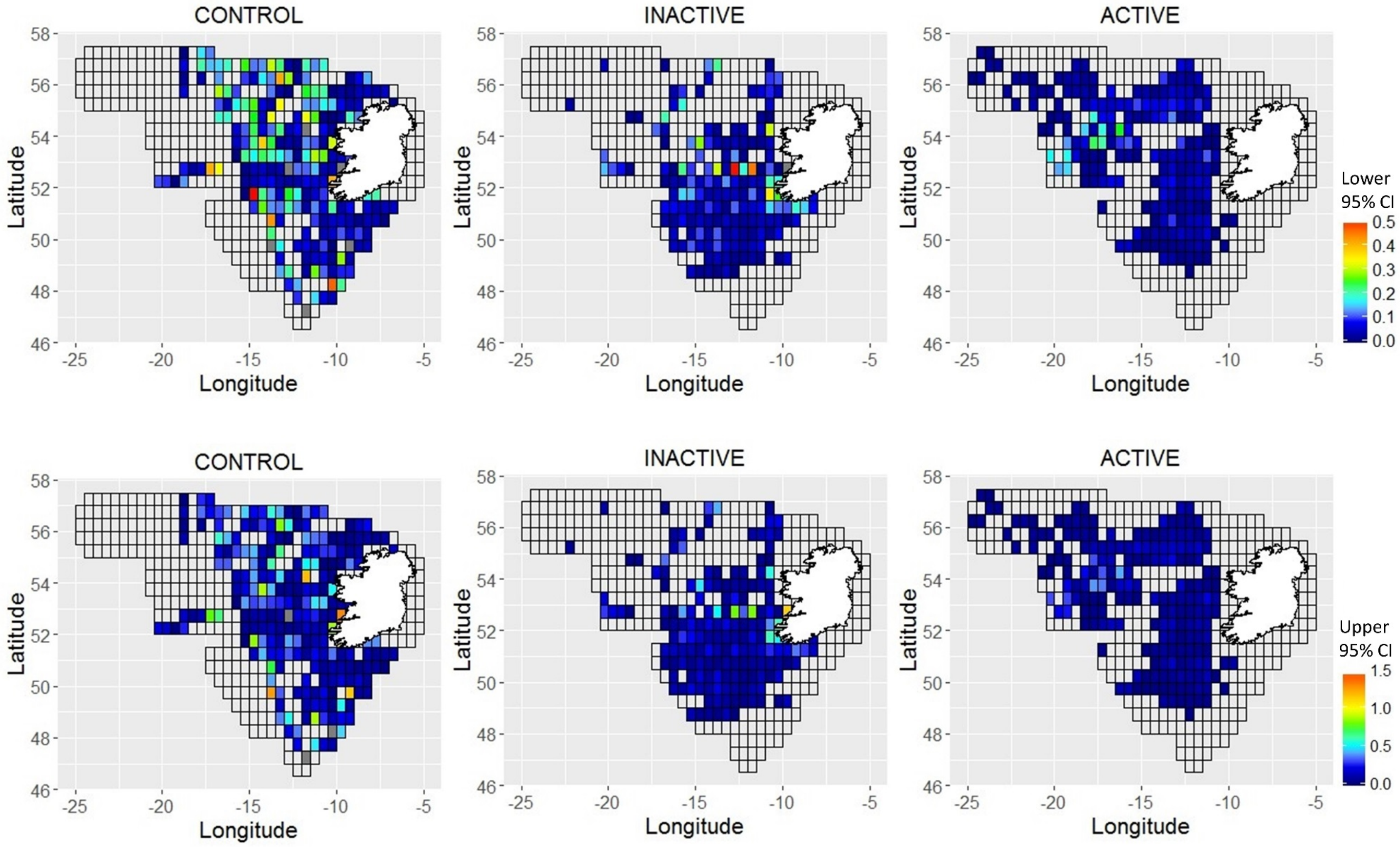

Fig. S7: Model predicted 95% confidence interval (CI) of sighting densities (per grid cell) for toothed whales in the control model (i.e. the effect of inactive and active seismic surveys compared to the control surveys).

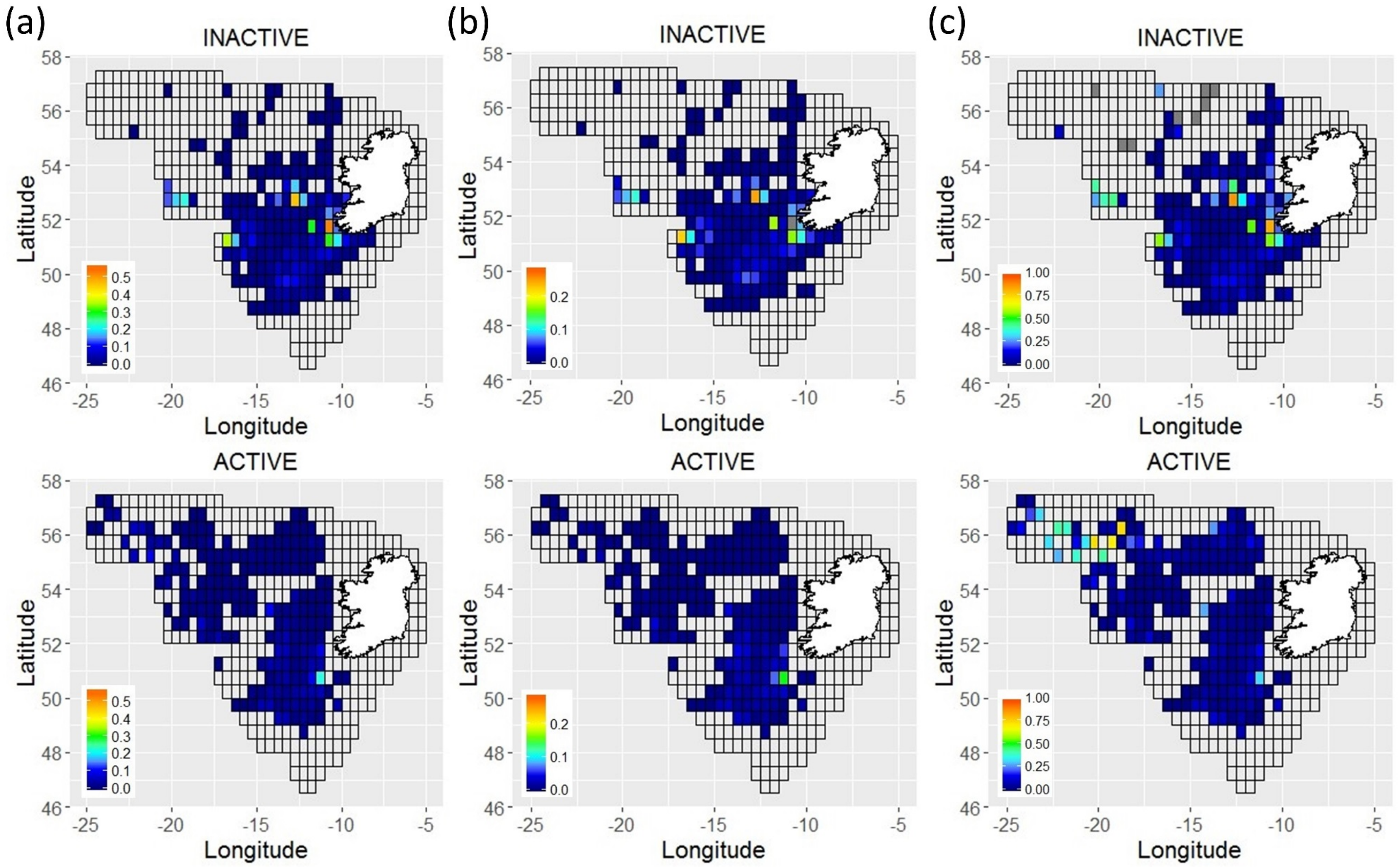

Fig. S8: Model predicted sighting densities per grid cell for baleen whales for seismic models (i.e. the effect of inactive compared to active seismic surveys), with (a) mean, (b) lower 95% confidence interval, and (c) upper 95% confidence interval.

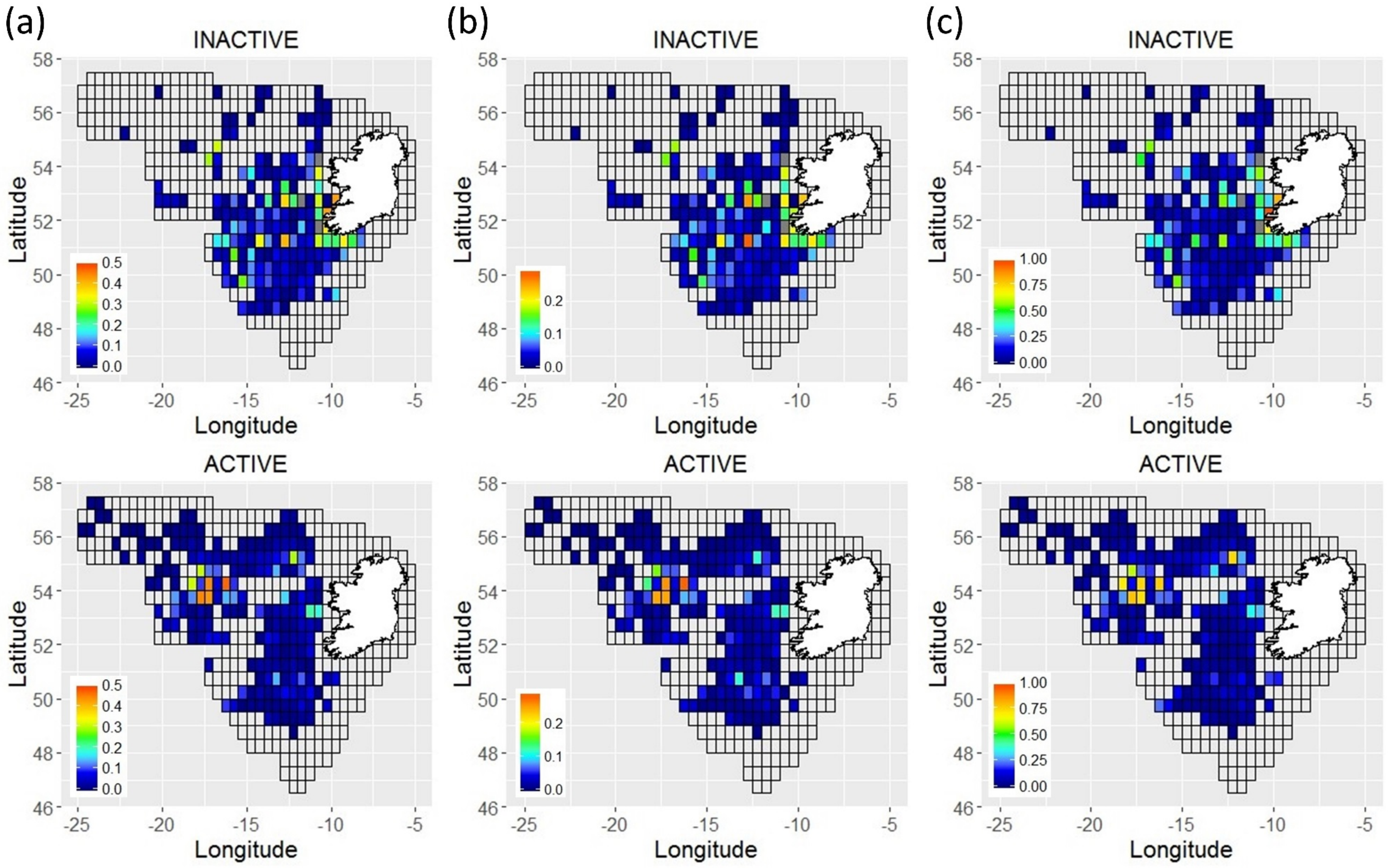

Fig. S9: Model predicted sighting densities per grid cell for toothed whales for seismic models (i.e. the effect of inactive compared to active seismic surveys), with (a) mean, (b) lower 95% confidence interval, and (c) upper 95% confidence interval.

Table S1: Seismic and control surveys included in the analysis

Survey ID	Survey Type		Year
S10_16	Seismic	CGG	2016
S09_16	Seismic	Woodside Energy	2016
S08_16	Seismic	Woodside Energy	2016
S07_15	Seismic	Searcher Seismic Pty Ltd	2015
S06_14	Seismic	Capricorn Ireland	2014
S05_14	Seismic	ENI Ireland	2014
S04_14	Seismic	Polarcus Multi-Client	2014
S03_14	Seismic	GeoPartners Ltd	2014
S02_13	Seismic	ENI Ireland	2013
S01_13	Seismic	Kosmos Energy Ireland	2013
C16_17	Control	Blue Whiting Acoustic Survey	2017
C15_17	Control	WESPAS	2017
C14_17	Control	Blue Whiting Acoustic Survey	2017
C13_16	Control	Haddock Trawl Survey	2016
C12_16	Control	MI Boarfish Survey	2016
C11_16	Control	MI Mackerel Egg Survey	2016
C10_16	Control	MI Mackerel Egg Survey	2016
C09_16	Control	MI Mackerel Egg Survey	2016
C08_16	Control	MI Mackerel Egg Survey	2016
C07_16	Control	MI Mackerel Egg Survey	2016
C06_16	Control	Trans-Atlantic Survey	2016
C05_16	Control	Trans-Atlantic Survey	2016
C04_16	Control	ROV Survey	2016
C03_15	Control	Trans-Atlantic Survey	2015
C02_15	Control	ROV Survey	2015
C01_15	Control	Groundfish Survey	2015

Table S2: Proportion of sightings for control, active seismic and inactive seismic surveys (with sighting sample size in brackets), for baleen and toothed whales. Most commonly recorded baleen and toothed whale species are highlighted with an asterisk (*).

	**Proportion of Sightings**		
**Mysticete Species (Baleen Whales)**	Control	Active	Inactive
Blue whale (*Balaenoptera musculus*)	0 (0)	0.03 (6)	0.05 (8)
Fin whale (*Balaenoptera physalus*)*	0.43 (62)	0.45 (98)	0.44 (77)
Sei whale (*Balaenoptera borealis*)	0.01 (2)	0.02 (4)	0.01 (1)
Humpback whale (*Megaptera novaeangliae*)	0.08 (11)	0.03 (7)	0.02 (4)
Minke whale (*Balaenoptera acutorostrata*)	0.31 (44)	0.05 (11)	0.11 (19)
Unidentified Mysticete Species	0.17 (24)	0.42 (93)	0.38 (66)
**Odontocete Species (Toothed Whales)**			
Harbour porpoise (*Phocoena phocoena*)	0.02 (13)	0 (0)	0 (0)
Bottlenose dolphin (*Tursiops truncatus*)	0.07 (39)	0.05 (15)	0.07 (23)
Common dolphin (*Delphinus delphis*)*	0.43 (236)	0.49 (154)	0.50 (154)
Cuvier's beaked whale (*Ziphius cavirostris*)	<0.01 (1)	0 (0)	<0.01 (1)
Blainville's beaked whale (*Mesoplodon densirostris*)	<0.01 (1)	0 (0)	0 (0)
Sowerby's beaked whale (*Mesoplodon bidens*)	<0.01 (1)	<0.01 (1)	0 (0)
Striped dolphin (*Stellena coeruleoalba*)	0.02 (10)	0.02 (7)	0.01 (3)
Risso's dolphin (*Grampus griseus*)	0.01 (7)	0.01 (3)	0.01 (3)
White-beaked dolphin (*Lagenorhynchus albirostris*)	0.02 (12)	0 (0)	<0.01 (1)
White-sided dolphin (*Lagenorhynchus acutus*)	0.03 (16)	0 (0)	0 (0)
Sperm whale (*Physter macrocephalus*)	0.07 (41)	0.05 (17)	0.05 (15)
Killer whale (*Orcinus orca*)	<0.01 (2)	<0.01 (1)	0.01 (3)
False killer whale (*Pseudorca crassidens*)	<0.01 (1)	0 (0)	0 (0)
Pilot whale (*Globicephala melas*)	0.17 (92)	0.16 (51)	0.17 (52)
Unidentified Odontocete Species	0.14 (76)	0.20 (63)	0.18 (55)
